# Adherence to the Dutch Breast Cancer Guidelines for Surveillance in Breast Cancer Survivors: Real-World Data from a Pooled Multicenter Analysis

**DOI:** 10.1093/oncolo/oyac126

**Published:** 2022-08-13

**Authors:** Teresa Draeger, Vinzenz Voelkel, Kay Schreuder, Jeroen Veltman, Anneriet Dassen, Luc Strobbe, Harald J Heijmans, Ron Koelemij, Catharina G M Groothuis-Oudshoorn, Sabine Siesling

**Affiliations:** Tumor Center Regensburg/University of Regensburg, Institute for Quality Control and Health Services Research, Regensburg, Germany; Department of Health Technology and Services Research, Technical Medical Centre, University of Twente, Enschede, The Netherlands; Tumor Center Regensburg/University of Regensburg, Institute for Quality Control and Health Services Research, Regensburg, Germany; Department of Health Technology and Services Research, Technical Medical Centre, University of Twente, Enschede, The Netherlands; Department of Research and Development, Netherlands Comprehensive Cancer Organisation (IKNL), Utrecht, The Netherlands; Department of Radiology, Ziekenhuisgroep Twente, Almelo, The Netherlands; Department of Surgery, Medisch Spectrum Twente, Enschede, The Netherlands; Department of Surgery, Canisius Hospital, Nijmegen, The Netherlands; Department of Surgery, Ziekenhuisgroep Twente, Hengelo, The Netherlands; Department of Surgery, Antonius Ziekenhuis, Nieuwegein, The Netherlands; Department of Health Technology and Services Research, Technical Medical Centre, University of Twente, Enschede, The Netherlands; Department of Health Technology and Services Research, Technical Medical Centre, University of Twente, Enschede, The Netherlands; Department of Research and Development, Netherlands Comprehensive Cancer Organisation (IKNL), Utrecht, The Netherlands

**Keywords:** breast cancer, follow-up, health services research, guideline adherence, daily clinical practice

## Abstract

**Background:**

Regular follow-up after treatment for breast cancer is crucial to detect potential recurrences and second contralateral breast cancer in an early stage. However, information about follow-up patterns in the Netherlands is scarce.

**Patients and Methods:**

Details concerning diagnostic procedures and policlinic visits in the first 5 years following a breast cancer diagnosis were gathered between 2009 and 2019 for 9916 patients from 4 large Dutch hospitals. This information was used to analyze the adherence of breast cancer surveillance to guidelines in the Netherlands. Multivariable logistic regression was used to relate the average number of a patient’s imaging procedures to their demographics, tumor–treatment characteristics, and individual locoregional recurrence risk (LRR), estimated by a risk-prediction tool, called INFLUENCE.

**Results:**

The average number of policlinic contacts per patient decreased from 4.4 in the first to 2.0 in the fifth follow-up year. In each of the 5 follow-up years, the share of patients without imaging procedures was relatively high, ranging between 31.4% and 33.6%. Observed guidelines deviations were highly significant (*P* < .001). A higher age, lower UICC stage, and having undergone radio- or chemotherapy were significantly associated with a higher chance of receiving an imaging procedure. The estimated average LRR-risk was 3.5% in patients without any follow-up imaging compared with 2.3% in patients with the recommended number of 5 imagings.

**Conclusion:**

Compared to guidelines, more policlinic visits were made, although at inadequate intervals, and fewer imaging procedures were performed. The frequency of imaging procedures did not correlate with the patients’ individual risk profiles for LRR.

## Teaser

Regular follow-up after treatment for breast cancer is important for early detection of recurrence. This article evaluates adherence to the current Dutch breast cancer guidelines for follow-up care after curative breast cancer treatment in daily clinical practice.

**Implications for Practice**

In daily clinical practice, breast cancer follow-up schedules deviate significantly from guideline recommendations; patients obtain more policlinic visits, although at inadequate intervals, and less imaging procedures than formally necessary. Deviation from guidelines does not take individual risk profiles into account and seems to occur at random. Therefore, consideration should be given to changing guideline recommendations toward follow-up schedules based on reliable individual risk-estimations, provided by validated prediction tools like INFLUENCE. The burden on the health care system could be reduced if this could increase patient adherence to their schedules.

## Introduction

Breast cancer is the most frequent malignancy among women in the Netherlands and worldwide.^1-3^ During the last decades, the overall survival rates after diagnosis have been increasing considerably due to early detection and improved treatment strategies.^4-7^ In the Netherlands, 85% of the women diagnosed with breast cancer are still alive 5 years after diagnosis and 76% survive at least 10 years on average.^2^ After finalizing primary treatment, breast cancer patients receive follow-up care which focusses on the detection of locoregional recurrences (LRR) and second primary contralateral breast cancer to improve survival.^8,9^ According to the current Dutch breast cancer guidelines, every woman who has undergone curative treatment for breast cancer is eligible for follow-up, which consists of annual follow-up visits and imaging procedures like mammographies or MRIs for patients without a genetic predisposition.^10^ The Dutch breast cancer guidelines were introduced in 2002 and updated to Version 2.0 in 2012. In the course of this update, some changes concerning follow-up recommendations were made. During the last revision in 2020, follow-up recommendations remained unchanged.

Only a limited number of studies examined the actual usage of mammographies and other diagnostic procedures in breast cancer survivors. An earlier but considerably smaller study on guidelines adherence in the Netherlands revealed that the number of consultations exceeded the recommended number in a case where radiation therapy as part of the primary treatment. On the other hand, it has been shown that less follow-up mammographies than recommended were performed.^11^ Studies from other countries likewise found the actual surveillance care patterns to differ from guidelines and showed a general underutilization of mammographies in breast cancer follow-up.^12-14^ Moreover, some studies observed a steady decline in the number of performed mammographies as the time after diagnosis increased.^15-17^ Different patient and primary treatment characteristics like age or adjuvant radiation therapy seem to influence the utilization of subsequent mammographies. Apart from that, there are also women who do not receive any follow-up at all.^18^

Using a large multicenter cohort from the Netherlands, the main aim of this study was to evaluate the adherence to the Dutch breast cancer guidelines for follow-up care after curative breast cancer treatment in daily clinical practice. It is supposed to provide an overview of the number of policlinic visits and applied imaging procedures in the first 5 years following diagnosis. Additionally, the association of different patient, tumor, and treatment characteristics with the application of follow-up imaging should be investigated. Finally, the LRR risk for every patient was estimated based on the INFLUENCE-nomogram, a prediction model for breast cancer survivors,^19^ and correlated with the observed utilization of diagnostic imaging to determine whether deviation from the guidelines is correlated with the estimated risk of LRR of an individual patient.

## Patients and Methods

### Study Population

For this retrospective multicenter cohort study, all female breast cancer patients from 4 large breast cancer centers in the Netherlands (Canisius Wilhelmina Hospital, Nijmegen, St. Antonius Hospital, Nieuwegein, Medisch Spectrum Twente, Enschede, Ziekenhuisgroep Twente, Almelo/Hengelo) who underwent curative unilateral surgery for invasive breast cancer (ICD-10 C50) diagnosed between 2006 and 2017, without distant metastases, synchronous, or previous breast tumors were eligible for inclusion in the analysis. Curative resection was defined as the surgical removal of the primary carcinoma without macroscopic residual tumor.

### Data Collection

The *Performation* database *(Performation.com)* contains detailed Information about aftercare diagnostics provided to these patients between 2009 and 2019. At the patient level, this data was linked to the corresponding demographics, tumor, and treatment characteristics provided by the Netherlands Cancer Registry (NCR). The NCR is a nationwide population-based registry, that has been systematically collecting data on all newly diagnosed malignancies in the Netherlands since 1989. Information on each patient has been gathered from the patient files by specially trained registration clerks.

### Definition of Outcomes

Based on the Dutch breast cancer guidelines, the first follow-up visit should be performed approximately one year after the last imaging procedure before surgery. Thereafter, an annual follow-up interval is recommended.^10^ Usually, a follow-up visit consists of a policlinic visit (defined as a visit linked to the underlying breast cancer diagnosis ICD-10 C50 in either the department of surgical oncology, radiation therapy, plastic surgery, or medical oncology) and an imaging procedure (usually a mammography, on indication supplemented with MRI or sonography).

To account for the variations which will be seen in daily clinical practice from which our data originates, it was decided to define the 5 follow-up years dictated by the guidelines in a flexible way into follow-up period: any examination, mammography, MRI, and/ or sonography performed between months 5-15 after surgery was defined as “follow-up 1”; correspondingly, diagnostic imaging procedures performed between 16 and 28 months were regarded as follow-up 2, etc. (follow-up 3: 29-41 months, follow-up 4: 42-54 months, follow-up 5: 55-67 months). At an individual level, guideline adherence was defined as taking up at least one follow-up visit and at least one imaging procedure in each of the 5 potential follow-up periods.

### Statistical Analysis

For all 5 recommended follow-up visits after diagnosis, the average number of policlinic visits, mammographies, MRIs, and sonographies per patient was calculated. Regarding the imaging procedures, patients were divided into 3 groups: “Mammography only” (MO-patients), “any Combination of mammography/MRI/ sonography” (MMS-patients), or “no Imaging” (NI-patients). Only those patients with a sufficient follow-up time and existing information for the corresponding time period were included in the analyses. Since patients could only be included in analyses corresponding to their survival- and follow-up period, a special subgroup analysis concentrated exclusively on patients with a complete follow-up time of at least 5 years and information about the performed diagnostic procedures for all 5 follow-up periods. To statistically assess guideline adherence, Fisher’s exact test was used.

Multivariable logistic regression analyses were performed to examine which factors were associated with a high or low chance of obtaining at least one follow-up imaging procedure per year. For this purpose, missing values concerning patient, tumor, and treatment characteristics were estimated using a chained equation approach for multiple imputations.^20-22^ It was assumed that missing values occurred randomly. The logistic regression was performed on 5 imputed datasets and pooled by using Rubin’s rules to obtain the final results, which were additionally compared to a parallel complete case analysis.

Furthermore, to determine whether deviation from guidelines was related to patients’ individual 5-year risk for a locoregional recurrence (LRR), this risk was estimated using the INFLUENCE-nomogram, a *Time-Dependent Prognostic Nomogram for the Estimation of Annual Risk of Locoregional Recurrence in Early Breast Cancer Patients,*^6^ which has been developed at the University of Twente in cooperation with the Dutch cancer registry IKNL. This risk prediction tool has been updated in 2021^23^ and is available online (https://www.evidencio.com/models/show/721). Only patients without missing data concerning nomogram variables (age, tumor size, nodal involvement, grade, estrogen/progesterone receptor (ER/PR) status, multifocality, radiotherapy, chemotherapy, and endocrine therapy) were included in this analysis. In a second step, the mean 5-year overall LRR risks of patients without any follow-up imaging at all and patients with regular follow-up imaging were compared.

All performed significance tests were 2-sided with a significance level of 0.05.

For the analyses IBM SPSS 25 (IBM Corp., SPSS for Windows, Armonk, NY, USA), R version 3.5.1 (R Foundation for Statistical Computing, Vienna, Austria; http://www.R-project.org/) and the R package “MICE” (https://CRAN.R-project.org/package=mice) were used.

## Results

In total, 9916 patients were included in this study. The number of cases contributed by the different hospitals was 1896, 2551, 2422, and 3047, respectively. The demographical, pathological, and treatment-associated features of the included patients are displayed in Table 1, both in total and at the hospital-level.

**Table 1.** Characteristics of the included breast cancer patients, stratified for the hospital (anonymized).

	Hospital 1		Hospital 2		Hospital 3		Hospital 4		Overall	
	n	%	n	%	n	%	n	%	n	%
Age group, years										
<50	614	24.1	675	27.9	413	21.8	675	22.2	2377	24.0
50-59	728	28.5	718	29.6	491	25.9	781	25.6	2718	27.4
60-69	673	26.4	640	26.4	543	28.6	870	28.6	2726	27.5
≥70	536	21.0	389	16.1	449	23.7	721	23.7	2095	21.1
Histological type										
Mixed	86	3.4	76	3.1	117	6.2	180	5.9	459	4.6
Ductal	2014	78.9	1860	76.8	1377	72.6	2291	75.2	7542	76.1
Lobular	308	12.1	312	12.9	234	12.3	333	10.9	1187	12.0
Other	143	5.6	174	7.2	168	8.9	243	8.0	728	7.3
Grading										
1	512	20.1	725	29.9	521	27.5	788	25.9	2546	25.7
2	1074	42.1	887	36.6	809	42.7	1327	43.6	4097	41.3
3	652	25.6	603	24.9	420	22.2	723	23.7	2398	24.2
Unknown	313	12.3	207	8.5	146	7.7	209	6.9	875	8.8
UICC stage										
I	1294	50.7	1161	47.9	885	46.7	1438	47.2	4778	48.2
II	963	37.7	993	41.0	791	41.7	1212	39.8	3959	39.9
III	294	11.5	268	11.1	220	11.6	397	13.0	1179	11.9
Tumor size (mm)										
<20	1784	69.9	1580	65.2	1161	61.2	1855	60.9	6380	64.3
20-50	633	24.8	697	28.8	635	33.5	997	32.7	2962	29.9
>50	92	3.6	106	4.4	73	3.9	143	4.7	414	4.2
Unknown	42	1.6	39	1.6	27	1.4	52	1.7	160	1.6
Multifocality										
No	2072	81.2	1977	81.6	1565	82.5	2480	81.4	8094	81.6
Yes	446	17.5	428	17.7	324	17.1	544	17.9	1742	17.6
Unknown	33	1.3	17	0.7	7	0.4	23	0.8	80	0.8
Lymph nodes										
0	1624	63.7	1577	65.1	1177	62.1	1863	61.1	6241	62.9
1-3	722	28.3	662	27.3	549	29.0	887	29.1	2820	28.4
>3	168	6.6	175	7.2	144	7.6	275	9.0	762	7.7
Unknown	37	1.5	8	0.3	26	1.4	22	0.7	93	0.9
Hormone receptor status (ER, PR)										
Negative	396	15.5	328	13.5	273	14.4	478	15.7	1475	14.9
Positive	2126	83.3	2078	85.8	1607	84.8	2546	83.6	8357	84.3
Unknown	29	1.1	16	0.7	16	0.8	23	0.8	84	0.8
Type of surgery										
Breast conserving surgery	1576	61.8	1696	70.0	900	47.5	1285	42.2	5457	55.0
Mastectomy	975	38.2	725	29.9	996	52.5	1761	57.8	4457	44.9
Unknown	0	0.0	1	0.0	0	0.0	1	0.0	2	0.0
Chemotherapy										
No	1454	57.0	1260	52.0	1122	59.2	1980	65.0	5816	58.7
Yes	1097	43.0	1162	48.0	774	40.8	1067	35.0	4100	41.3
Anti-hormonal therapy										
No	1113	43.6	1042	43.0	786	41.5	1563	51.3	4504	45.4
Yes	1438	56.4	1380	57.0	1110	58.5	1484	48.7	5412	54.6
Radiotherapy										
No	729	28.6	504	20.8	704	37.1	1257	41.3	3194	32.2
Yes	1822	71.4	1918	79.2	1192	62.9	1790	58.7	6722	67.8
Total	2551	100.0	2422	100.0	1896	100.0	3047	100.0	9916	100.0

**Figure 1.**
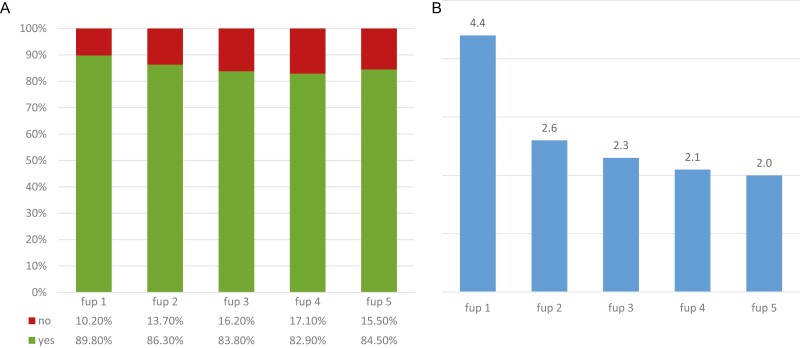
**(A):** Share of patients with at least one policlinic visit per follow-up interval, *n* = 9916, fup = follow up. **(B):** Mean number of policlinic visits per patient and follow-up interval (*n* = 9916).

There is a slight decline over time concerning policlinic visits: In the first follow-up period, 89.8% of the patients are seen at least once; the mean number of policlinic contacts per patient in this interval is 4.4. The rate of patients with at least one contact diminishes to 83.8% with a mean number of 2.3 visits in the third follow-up period. After that, the rate of patients with at least one policlinic contact remains almost constant at 82.9% in the fourth and 84.5% in the fifth period with a corresponding number of 2.1 and 2.0 visits per patient (Fig. 1a, 1b).

When only patients with a follow-up time of at least 5 years (*n* = 2160) are included in the analyzes, the results are quite comparable: The rate of patients with at least one policlinic visit per follow-up period is almost constant, ranging between 84.2% in the first and 83.4% in the fifth follow-up period with an isolated peak of 89.1% in the second period (Supplementary Fig. S1a). However, in this subgroup analysis, the number of policlinic visits per patient is also decreasing from 4.8 in the first follow-up period to 2.1. in the fifth (Supplementary Fig. S1b). Overall, 63.6% of the patients received at least one policlinic visit in each of the 5 follow-up periods, as dictated by the guidelines, and 20.1% of patients in 4 of 5 follow-up periods. A total of 2.1% of the patients had no follow-up visit at all. According to Fisher’s exact test, the observed guidelines deviation was highly significant (*P* < .001).

**Figure 2.**
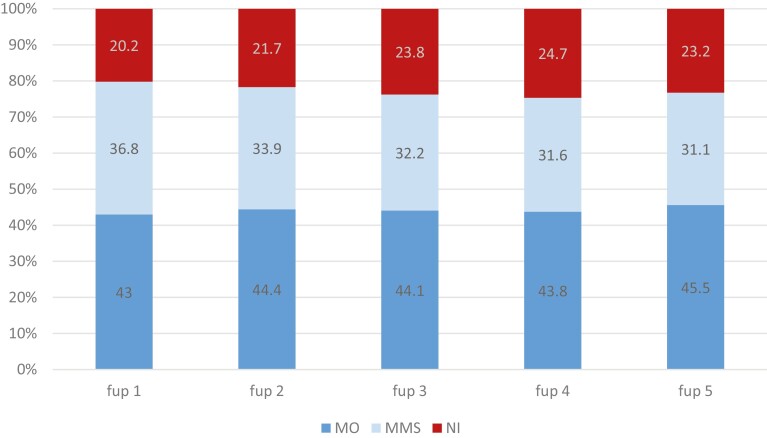
Distribution of applied imaging procedures stratified for follow-up intervals: mammography only (MO), any combination of mammography/MRI/ sonography (MMS), or no imaging (NI). (*n* = 9916), fup = follow up.

The rate of patients with at least one mammography conducted per follow-up period is relatively constant and ranges between 43.0% in the first and 45.5% in the fifth follow-up period; the rate of patients who received a combination of different imaging procedures like mammography, MRI, and sonography declines from 36.8% in the first to 31.1% in the fifth follow-up period (Fig. 2). The share of patients who obtained no imaging procedure in a follow-up period is relatively constant and ranges between 21.7% in the first and 24.7% in the fourth follow-up period.

The situation in the 5-year follow-up patients’ subgroup is quite comparable (Supplementary Fig. S2). Overall, 56.9% of the patients received at least one imaging procedure in each of the 5 follow-up periods, which is equivalent to full guidelines adherence, 21.9% of patients in 4 of 5 follow-up periods. A total of 7.2% of the patients had no imaging procedure at all within 5 years. The rate of non-adherence to guidelines for imaging/policlinic was statistically significant (*P* < .001).

Several factors are significantly associated with the chance to receive at least one follow-up imaging in each of the 5 follow-up periods. According to the logistic regression analysis pooled over 5 multiply imputed datasets, patients with higher age (Ref.: <50; 50-59: OR 1.77, *P* = .003; 60-69: OR 2.14, *P < .*001; ≥ 70: OR 1.67, *P = .*08) and patients with lower UICC stage (Ref.: UICC I; UICC II: OR 1.23, *P = .*32; UICC III: OR 0.48, *P = .*04) were more likely to obtain an imaging procedure. Patients who received radiation therapy (OR 4.60, *P < .*001) or chemotherapy (OR 2.21, *P < .*001) did also have a higher chance of imaging procedures during follow-up. Other patient or treatment characteristics did not have a significant influence on the chances of receiving an imaging procedure (Table 2).

**Table 2.** Multivariable logistic regression on the chance to obtain at least one imaging procedure in all 5 follow-up intervals in patients with a complete 5-year follow-up (*n* = 2160).

	OR	*P*-value
Age group, years		
<50	Ref.	
50-59	1.77	**.03**
60-69	2.147	**< .001**
≥70	1.67	.08
Histological type		
Mixed	Ref.	
Ductal	1.20	.60
Lobular	1.27	.58
Other	2.42	.11
Grading		
1	Ref.	
2	1.07	.80
3	0.75	.33
UICC stage		
I	Ref.	
II	1.24	.32
III	0.48	**.04**
Multifocality		
No	Ref.	
Yes	1.03	.87
Hormone receptor status (ER, PR)		
Negative	Ref.	
Positive	1.29	.47
Hospital		
1	Ref.	
4	1.06	.73
Type of surgery		
BCS	Ref.	
MAST	0.77	.45
Chemotherapy		
No	Ref.	
Yes	2.21	<.001
Anti-hormonal therapy		
No	Ref.	
Yes	0.73	.23
Radiotherapy		
No	Ref.	
Yes	4.60	**<.001**

Abbreviations: ER, estrogen; PR, progesterone; BCS, breast conserving surgery; MAST, mastectomy.

Regular follow-up imaging correlates inversely with the estimated risk of LRR within 5 years after the end of treatment: the mean risk of a patient who did not receive any follow-up imaging at all was 3.5% (median 2.4%, interquartile range, IQR: 1.6-4.4%) vs. 2.8% (median 2.0%, IQR: 1.2-3.6%) in patients with 1-4 imaging procedures in 5 years and 2.3% (median 1.6%, IQR: 1.1-2.5%) in patients with the recommended, guidelines-adherent number of 5 imaging procedures.

## Discussion

The present study aims to evaluate adherence to the Dutch breast cancer guidelines for follow-up care after curative breast cancer treatment in daily clinical practice. The observed number of performed follow-up visits and imaging procedures differs significantly from the Dutch breast cancer guidelines. Whereas more policlinic visits as recommended are scheduled, a moderate underutilization of imaging procedures has been observed, which increases as time after primary treatment passes. Whether a patient receives regular imaging procedures correlates significantly with advancing age, lower UICC stage, and improved status after radiation or chemotherapeutic treatment. So far, these findings are entirely congruent with existing evidence on the topic from the Netherlands and other countries.^11-17^ In addition to this, another remarkable finding of the present study is that patients with less imaging procedures on average had a higher risk of LRR, estimated by INFLUENCE, a comprehensive risk prediction tool developed in cooperation with the Dutch cancer registry IKNL.

To our knowledge, this is the largest retrospective cohort study analyzing the actual utilization of breast cancer follow-up services, reflecting guideline implementation in terms of policlinic visits and imaging procedures in the Netherlands. The remarkable sample size of almost 10 000 patients from different regions in the Netherlands, treated in 4 dedicated breast centers, is a considerable strength of this study. The study cohort can be seen as high representative of the whole country and gives a reliable insight into adherence to current guidelines—or its lack.

Nonetheless, some limitations must be considered when interpreting the results presented in this study. Unfortunately, information on nononcologic comorbidities was not available, which might also influence the participation in follow-up. Furthermore, we did not have information for the entire follow-up period of 5 years for every patient. To avoid selection bias, we decided against excluding these patients; by using a flexible approach, taking into account differing lengths, starting-, and end-points of a patient’s follow-up, it was possible to include a maximum number of patients into the analysis at a specific time-interval of the follow-up period without compromising the analyses on other intervals.

Like in earlier studies on the topic from the Netherlands^11,24,25^ and Canada,^26^ we observed more policlinic contacts than necessary during the follow-up period. It has been shown that the follow-up frequency increases with the number of medical disciplines involved. On the other hand, Lu et al^27^ and Montgomery et al^28^ demonstrated that physical examination plays only a small role in the early detection of locoregional recurrences and second primary breast cancer. Based on these findings, the Dutch Breast Cancer guideline’s recommendations concerning policlinic visits for the first year after diagnosis were changed in 2012. According to the previous version of the guidelines issued in 2002, one policlinic visit every 3 months during the first year of follow-up had been recommended. The new version 2.0, issued in February 2012, states that women should receive only one policlinic visit in each follow-up year.^10^ As of today, these recommendations are still valid. A certain share of patients in our study cohort still received follow-up according to the now outdated, earlier version of the guidelines, which partly explains the high number of policlinic visits observed in the first follow-up period. Furthermore, it can be assumed that the practical implementation of new guidelines takes some time. However, there could also be other important reasons for the observed policlinic overuse: Patients and caregivers might regard one policlinic visit per year as insufficient to receive or provide adequate psychosocial care and monitor long-term side-effects of primary treatment, especially during the first follow-up year.^29,30^ It has been shown that continuous monitoring and treatment of impaired quality of life is associated with significant positive effects for the patients.^31^ This topic should receive more attention in the future.

Concerning the improvement of survival rates and early detection of tumor recurrences and second contralateral primary tumors, diagnostic imaging is unarguably the most essential part of follow-up care. The present study revealed interesting correlations between patient features and follow-up patterns. For example, patients having undergone radiation therapy turned out to have a higher chance of receiving imaging procedures during follow-up. A reason for this might be because these patients are treated by 2 different specialists, both involved in the follow-up process, as seen in previous research.^26^ However, a considerable share of patients with a curatively resected breast tumor obtains less than recommended imaging procedures or even none at all. Unfortunately, we were not able to directly inquire about the reasons for the omission of follow-up imaging, which is a limitation of this study. However, in one of the few existing studies on this topic, Wirtz et al found that “important subgroups of women are at high risk for non-adherence to surveillance recommendations, even among younger breast cancer survivors.“^18^ Moreover, Guarneri et al showed that the use of mammography is subject to substantial regional variability and, in general, lower than expected. Non-adherence due to a deficient awareness of the disease may be one reason for this. Some women might be afraid of a recurrence detected during follow-up and therefore choose not to show up. This could also explain why adherence to guidelines concerning imaging procedures is even lower than that concerning policlinic visits in general. Whether this is due to the non-adherence of the clinician or the patients cannot be determined with the data available. Before this background, Freedman proposed “reframing discussions around surveillance mammography” and “taking into account life expectancy, the estimated risk for subsequent in-breast events, and patient preferences”.^32^ Currently, we are about to take the next step in this direction. Great efforts are taken to personalize medical care. The INFLUENCE nomogram used in this study was designed to estimate a patient’s individual risk for a locoregional breast cancer recurrence and could be used to optimize follow-up allocation.^19^ We hypothesize that, along with other factors, patient awareness of individual recurrence risk could contribute to adherence to regular follow-up imaging procedures. Furthermore, personalized follow-up schemes based on the individual risk estimations for breast cancer LRR could decrease the number of follow-up visits.^33^

Another reason for the observed underutilization of imaging might be that some of the patients switched to the national screening program. In the Netherlands, women aged between 50 and 74 years old are invited biannually for a screening mammography. A previous study revealed that only 4% of the patients went to both the follow-up and the screening program within 5 years of their treatment.^34^ Besides, we do not know whether patients developed a recurrence during the follow-up time. Taking into account the average risk of LRR of 2.6% in the Netherlands within 5 years,^19^ approximately 250 patients in the study group ought to have developed an LRR and consequently dropped out of the follow-up program. The lower chance of receiving an annual imaging procedure in UICC stage III might be influenced by the higher risk of developing metachronous distant metastases in comparison to lower stages.^35^ Data from our study seems to reveal that patients with a mastectomy also received less follow-up imaging than patients with a breast conserving therapy. Although this association was not significant, at first glance this is a plausible observation, since a mammography without remaining breast tissue is not possible. However, even patients with a mastectomy are still at risk of developing a second primary tumor in the contralateral breast, which could be detected by a mammography. Moreover, they can also suffer from an ipsilateral recurrence in the chest wall, which could be detected by sonography. Therefore, more in-depth research on this topic is warranted.

## Conclusion

In the large cohort from the Netherlands analyzed in this study, breast cancer follow-up deviated significantly from national guidelines. More policlinic visits and less imaging procedures than recommended were observed. The frequency of performed imaging procedures did not correlate with the patients’ individual risk profiles for LRR. Regular usage of risk prediction models could contribute to the personalization of follow-up schedules and improve compliance. Moreover, the burden on health care and costs could be reduced.

## Supplementary Material

oyac126_suppl_Supplementary_Material

## Data Availability

The data underlying this article will be shared on reasonable request to the corresponding author.
