# Supplementary material for: Adherence to the Dutch Breast Cancer Guidelines for Surveillance in Breast Cancer Survivors: Real-World Data from a Pooled Multicenter Analysis
Source: Oncologist. 2022 Aug 13;27(10):e766–73. doi: 10.1093/oncolo/oyac126 (PMC9526487; doi:10.1093/oncolo/oyac126)
Supplement: oyac126_suppl_Supplementary_Material [file oyac126_suppl_supplementary_material.docx]

Supplementary figure 1a. Share of patients with at least one policlinic visit per follow-up interval (complete five year follow-up cases only, n = 2,160)

Supplementary figure 1b. Mean number of policlinic visits per patient and follow-up interval (complete five year follow-up cases only, n = 2,160), fup = follow-up

Supplementary figure 2. Distribution of applied imaging procedures stratified for follow-up intervals: Mammography only (MO), any combination of mammography/ MRI/ sonography (MMS) or no imaging (NI). (complete five year follow-up cases only, n = 2,160), fup = follow-up
